# The genetic structure of a *Venturia inaequalis* population in a heterogeneous host population composed of different *Malus* species

**DOI:** 10.1186/1471-2148-13-64

**Published:** 2013-03-12

**Authors:** Thibault Leroy, Christophe Lemaire, Frank Dunemann, Bruno Le Cam

**Affiliations:** 1 Université d’Angers, IRHS, PRES UNAM, SFR QUASAV, Boulevard Lavoisier, Angers, 49045 France; 2 INRA, IRHS, PRES UNAM, SFR QUASAV, Rue Georges Morel, Beaucouzé, 49071 France; 3 Agrocampus Ouest, IRHS, PRES UNAM, SFR QUASAV, Rue Le Nôtre, F-49045 Angers, France; 4 Julius Kühn Institute (JKI), Federal Research Centre for Cultivated Plants, Institute for Breeding Research on Horticultural and Fruit Crops, Erwin-Baur-Strasse 27, 06484, Quedlinburg, Germany

**Keywords:** Gene flow, Isolation-by-distance (IBD), Apple scab, Adaptation, Spatial genetic structure

## Abstract

**Background:**

Adaptation, which induces differentiation between populations in relation to environmental conditions, can initiate divergence. The balance between gene flow and selection determines the maintenance of such a structure in sympatry. Studying these two antagonistic forces in plant pathogens is made possible because of the high ability of pathogens to disperse and of the strong selective pressures exerted by their hosts. In this article, we analysed the genetic structure of the population of the apple scab fungus, *Venturia inaequalis*, in a heterogeneous environment composed of various *Malus* species. Inferences were drawn from microsatellite and AFLP data obtained from 114 strains sampled in a single orchard on nine different *Malus* species to determine the forces that shape the genetic structure of the pathogen.

**Results:**

Using clustering methods, we first identified two specialist subpopulations: (i) a virulent subpopulation sampled on *Malus* trees carrying the *Rvi6* resistance gene; and (ii) a subpopulation infecting only *Malus* trees that did not carry this resistance gene. A genome scan of loci on these two subpopulations did not detect any locus under selection. Additionally, we did not detect any other particular substructure linked to different hosts. However, an isolation-by-distance (IBD) pattern at the orchard scale revealed free gene flow within each subpopulation.

**Conclusions:**

Our work shows a rare example of a very strong effect of a resistance gene on pathogen populations. Despite the high diversity of *Malus* hosts, the presence of *Rvi6* seems sufficient to explain the observed genetic structure. Moreover, detection of an IBD pattern at the orchard scale revealed a very low average dispersal distance that is particularly significant for epidemiologists and landscape managers for the design of scab control strategies

## Background

The diversification of pathogens on different hosts is commonly thought to arise via new adaptations in response to the disruptive selection exerted by host defences or non-host resistances. The “Red Queen’s race” hypothesis [1] describes the coevolution of hosts and pathogens in natural ecosystems. In agro-ecosystems, higher homogeneity and density of hosts, coupled with a low host species diversity, are expected to induce quick adaptive changes in pathogens [2]. Indeed, in pathogens, higher fecundity associated with large effective sizes and shorter generation times enhances the rise of new mutants able to settle on new hosts [3-6]. Adaptive mutations toward virulence can lead to founder effects that will remain detectable over the years only if gene flow with the resident population is prevented. In sympatry, high selection pressure experienced by the pathogen or strong assortative mating are required to maintain population structure over time by limiting the homogenising effects of gene flow between strains adapted to different hosts [7,8]. In pathogens that mate within their hosts, adaptation can induce a strong and stable reproductive isolation between populations that facilitates the maintenance of genetic differentiation [7,8]. Since an agricultural landscape can be seen as a mosaic of crops with multiple cultivars carrying different resistance traits, both adaptation and gene flow processes are expected to shape the genetic structure of pathogen populations. We can therefore ask if, as a result of these processes, a landscape constituted by different hosts will produce a mosaic of different pathogen populations, each specialised on a host species.

Short distances between plants in fields permit the rapid spread of a pathogen and large population sizes [2]. Since most pathogens produce large amounts of descendants through highly efficient sexual and/or asexual offspring productions (e.g., [9,10]), detectable gene flow between populations on different neighbour hosts is expected [11], provided that appropriate tools are used. The two-dimensional stepping stone model [12], a system in which individuals stochastically diffuse within a lattice, should fit the spatial structure encountered in fields. In such a model, the probability of gene flow is dependent on the connectivity between demes. The relatedness between strains therefore decreases with geographical distance. No occurrence of an Isolation-By-Distance (IBD) pattern at a field scale (several hundreds of metres) has been reported to date in fungal pathogens since evidence of IBD implies short distance dispersal, which is not the general outcome assumed for pathogens able to produce small sexual propagules [11,13]. An IBD pattern over a heterogeneous landscape of host species would indicate that strains are generalists. Under this hypothesis, two strains isolated from neighbour demes that infect different host species are expected to be more closely related than two strains isolated from two distant demes, even if they infect the same host. We could then question whether it remains possible to maintain pathogen differentiations related to hosts in a heterogeneous environment.

In summary, it is assumed that two types of patterns of population genetic structure exist for pathogen populations: IBD or/and hierarchical structure. First, is it possible to observe a structure related to the hosts? And second, can an IBD pattern be detected, suggesting that gene flow is only driven by free migration?

*Venturia inaequalis* is an ascomycete fungus responsible for scab, a major apple disease in most areas of the world. The interaction between *Malus x domestica* and *V. inaequalis* fits the gene-for-gene model. In a gene-for-gene interaction, the product of a resistance gene in the host recognises an “effector” gene product in the pathogen and activates a defence reaction that completely prevents infection [14]. Numerous resistance genes providing resistance against *V. inaequalis* have been identified within *Malus* species [15]. The life cycle of *V. inaequalis* comprises both sexual and asexual phases of reproduction. Sexual mating occurs during the winter inside dead leaves in the litter layer, between strains of opposite mating types that have infected the same leaf. Zygotes undergo immediate meioses and yield haploid ascospores that are released to initiate new infections in spring that subsequently disseminate via asexually produced conidia. The pathosystem *Malus* spp.-*V. inaequalis* is suitable to study both gene flow and host adaptation. Epidemiological studies suggest that both types of propagules are dispersed over short distances (< 50 metres) [16,17], which makes an IBD pattern possible at the orchard scale. Additionally, *V. inaequalis* is described as a good model for host adaptation in plant pathogens [7] because mating only occurs between strains that are able to infect identical hosts, thus facilitating the maintenance of adaptations to a host when host ranges do not overlap [18,19]. *V. inaequalis* is a pathogen that is well-known for overcoming resistance genes introgressed into cultivars from *M. x domestica* germplasm and from wild genetic resources of *Malus*[15,20]. Moreover, population genetic studies have shown that apple resistance genes might induce specialisation in *V. inaequalis* populations, thus favouring host-related adaptations [18,20]. For example, in agro-ecosystems, the presence of the resistance gene *Rvi6* in apple divides *V. inaequalis* into two populations: one emerging - vir*Rvi6* – that infects *Rvi6* cultivars, and another - avr*Rvi6 -* that infects cultivars without this resistance gene [20]. To date, the population structure between avr*Rvi6* and vir*Rvi6* is maintained in agro-ecosystems, even when *Rvi6* and non-*Rvi6* cultivars are planted in the same orchards [18].

The aim of this study was to evaluate which antagonistic force, host-related adaptation (e.g., virulence toward the *Rvi6* resistance gene) or gene flow, shapes the *V. inaequalis* genetic structure within a genetically heterogeneous orchard consisting of different species of *Malus*. Our prediction was the following: in the absence of adaptation, an IBD pattern was expected at the orchard scale that would indicate free gene flow between strains, regardless of the host. Using polymorphism of microsatellite and AFLP markers, we infer the genetic structure of *V. inaequalis* populations, allowing us to address the following question: do host adaptations or gene flow shape the structure of *V. inaequalis* populations?

## Methods

### Fungal sampling and genotyping

**Figure 1:**
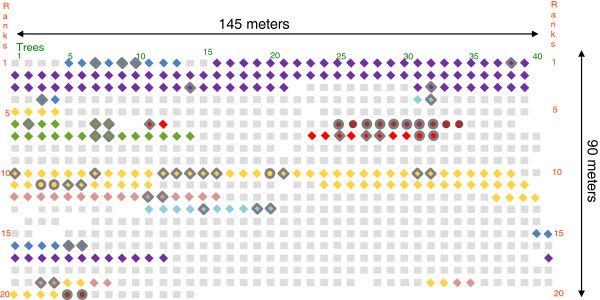
**The orchard map of *Malus *hosts where *V. inaequalis *strains were sampled.** Non-*Rvi6* host genotypes are represented by a diamond: *M. sieversii* (blue), *M. sylvestris* (purple), *M. coronaria* (green), *M. x purpurea* (light blue), *M. baccata* (yellow), *M. ioensis* (red) and *M. x zumi* (pink). *Rvi6* host genotypes are represented by a circle: *M. x floribunda* (brown), *M. baccata* (yellow) and *M. ioensis* (red). Other *Malus* species or hybrids not sampled (infected or not) are represented by grey squares.

Samples were isolated in a *Malus* orchard located in Dresden-Pillnitz (Saxony, Germany). This orchard has been free from fungicide treatment since it was planted in 1997. Within the orchard, *Malus* trees expressed a wide range of disease severity, from highly sensitive to fully resistant to *V. inaequalis*. A total of 114 strains derived from monoconidial isolates of *V. inaequalis* were sampled on 57 trees classified into eight groups: five from different species of *Malus* (*M. sieversii, M. sylvestris, M. baccata, M. ioensis* and *M. coronaria*) and three from hybrids of *Malus* (*M. x floribunda*, *M. x purpurea* and *M. x zumi*) (Figure 1, Additional file 1: Table S1).

Fungal DNA was extracted from monoconidial individuals using a phenol/chloroform protocol [21]*.* The individuals were genotyped at 11 microsatellite loci: *1tc1a*, *1tc1b*, *1tc1g*, *1aac3b*[22], *Vitcca7/P*, *Vitg11/70*, *Vicacg8/42*, *Viga7/116*, *Vica 9/152*, *Vica9/X*[23] and *M42*[19]. Individuals were also genotyped with an AFLP combination using the following selective primers: P*st*I primer + A (5^′^-GACTGAGTACGTGCAG**A-**3^′^) and M*sp*I primer + AA (5^′^-CGATGAGTCCTGAGCGG**AA**3^′^-). Both microsatellite and AFLP amplifications were performed according to a previously described protocol [20,24]. PCR products were scored against a fluorescently labelled size standard (400HD-rox for microsatellite and GS500-TAMRA for AFLP) in an ABI 3130 automated sequencer using Genemapper 4.0 software (Applied Biosystems, Foster City, CA, USA). Only fragments between 70 and 395 bp were scored. The presence or absence of a polymorphic marker was binary coded. The whole dataset composed of 11 SSR and 79 AFLP polymorphisms is available through the DRYAD data archive system (see the availability of supporting data). A clone-corrected dataset was used for all of the following tests.

### Plant material

The sampling strategy was established to select *V. inaequalis* strains from the greatest diversity of infected hosts available within the core orchard. Since the scab resistance gene *Rvi6* (previously named *Vf*[15]), introgressed from *M. x floribunda*, is known to exert a strong selective pressure on *V. inaequalis* populations in commercial orchards [18], we therefore investigated the presence/absence of the *Rvi6* locus within the genome of the sampled *Malus* accessions. All accessions were genotyped using three molecular markers tightly linked to the *Rvi6* locus on the apple linkage group 1 (LG 1) [25-28], except for the *M. ioensis* accession MAL330, which *Rvi6* allele sequences were already known (Dunemann, pers. comm.). An apple accession was classified as carrying the *Rvi6* locus when all three diagnostic alleles were detected (CH-Vf1: allele 159, AL07-SCAR: 480 bp fragment, Vfa2: 550 bp fragment) (Additional file 1: Table S1).

### Data analysis

#### Standard diversity indices, AMOVA and PCoA

Because samples were collected during the asexual stages of the *V. inaequalis* life cycle, isolates with exactly the same alleles at all loci were removed using Arlequin software, v. 3.11 [29] (clone-corrected dataset). A genetic distance matrix was created from the dataset under Genalex 6.1 [30]. An analysis of molecular variance (AMOVA) and a principal coordinates analysis (PCoA) were performed using this genetic distance matrix. The average gene diversity (H_d_;[31]) and the average number of alleles (A) were estimated from clone-corrected datasets using Arlequin [29].

#### Selection of neutral AFLP markers

In order to eliminate markers under selection for the subsequent genetic analyses, we performed an outlier detection using BayeScan software [32]. Using an estimation of locus-population specific F_ST_, this method determines a posterior probability of a locus to be under selection. We used the “decisive” threshold included on the user-friendly interface, which is equivalent to a 95% confidence interval.

#### Individual assignments

Individual assignments were performed using the Bayesian clustering method implemented in STRUCTURE v. 2.2.3 [33-35]. Admixture mode was used, and the Monte Carlo Markov Chain scheme was run for 500,000 iterations after an initial burn-in period of 50,000. We ran STRUCTURE for K clusters ranging from 1 to 8, and performed five repetitions to check for the convergence of likelihood values for each value of K. Evanno’s method, ΔK, was used to best estimate K [36,37]. This method was computed using the STRUCTURE HARVESTER programme, v. 0.56.3 [37].

Additional cluster analyses were performed using TESS, v. 2.3.1 [38-40]. TESS implements an individual-based spatially-explicit Bayesian algorithm, and uses a hidden Markov random field model to compute the proportion of individual genomes originating in K populations. The hidden Markov random field accounts for spatial connectivity and incorporates a decay of membership coefficient correlation with distance, which is a property similar to IBD. The algorithm was run with a burn-in period of 50,000 cycles and the estimation was performed using 100,000 additional cycles. We increased the maximum number of clusters from Kmax = 2 to Kmax = 8 (100 replicates for each value). We used 10% of simulations that minimised the Deviance Information Criterion (DIC) to obtain simulations that best fit the model for each K, and discarded 90 simulations. The ten retained simulations were subsequently analysed under CLUMPP, v. 1.1.2 [41], to average the estimated admixture coefficients. The averaged admixture coefficients were used as inputs for the spatial interpolation. Interpolations were performed using the R script [42] available with TESS software (see the TESS users’ manual).

#### Classic and partial mantel tests

The Meirmans procedure [43] was performed to test whether population structure detected by STRUCTURE was due to an IBD or to hierarchical clustering. Such an approach is based on computation of several independent Mantel tests using two different matrices and possibly a third matrix as a covariate for partial Mantel tests. The three different matrices are: (1) a genetic distance matrix; (2) a Euclidian geographic distance matrix; and (3) a matrix of cluster membership. The third matrix describes whether comparisons were made between individuals mainly assigned to the same cluster by STRUCTURE (1) or to different clusters (2). Comparing *r* values and the significance of the Mantel test (e.g., clusters and genetic distance vs*.* geographic and genetic distance) reveals whether the structure is mainly spatial or hierarchical. For details on the procedure, see [43]. These tests were performed using the VEGAN package [44] in R [42].

#### Testing for IBD patterns

The detection of IBD was performed using two methods. The first method is implemented in SPAGeDI software, v. 1.2 [45], and the second one uses variograms of gene diversity [46]. In an IBD pattern, kinship is expected to linearly decrease with geographic distance when the demes are connected according to a one-dimensional stepping stone (1D-SS) model, and to linearly decrease with the natural logarithm of distance according to a two-dimensional stepping stone (2D-SS) model [47,48]. Under an IBD model, an autocorrelogram shows a decreasing curve of the mean kinship between strains when the distance increases, whereas in a variogram of gene diversity, the curve represents the strain differences and is therefore expected to increase with distance. The gradual decrease of the relationship with distance between strains can be explained by limited migration abilities.

Using SPAGeDI, pairwise Loiselle estimators (F_ij_) of kinship [49] between individuals were estimated from all microsatellite and neutral AFLP loci. F_(d)_ statistics were calculated from the means of F_ij_ between pairs of individuals inside distance classes. Linear regression and pairwise Euclidian spatial distances between individuals were tested using a permutation procedure. Permutations were performed 20,000 times on localisations and 20,000 times on individuals. The autocorrelograms produced represent F_(d)_ plotted against the natural logarithm of distance (i.e., in a field, the 2D-SS model, a model where individuals move at random within a lattice [48], appears closer to reality).

Confirmations of the IBD tests were performed using variograms of gene diversity, as described by Wagner *et al*. [46].

$${\overset{⌢}{Y}}_{l}\left(r\right)=\sum_{k}\sum_{a<b}\frac{\chi_{\mathrm{ab}}^{\left(r\right)}}{2n_{r}}{\left(z_{\text{lka}}-z_{\text{lkb}}\right)}^{2}$$

Ninety-five percent confidence intervals were estimated by manually analysing 100 different permuted datasets and observing the variation range of ${\overset{⌢}{Y}}_{1}$ gene diversity for each r class. For each of the 100 datasets, we performed permutations of individuals: 500 within the vir*Rvi6* subpopulation and 1000 within the avr*Rvi6* subpopulation.

## Results

### The presence of the *Rvi6* resistance gene in *Malus* accessions divides the *V. inaequalis* population into two subpopulations

Among the 114 strains analysed, we observed 106 unique haplotypes based on 11 microsatellite loci and 79 AFLP markers (Additional file 1: Tables S2). The number of alleles at each microsatellite locus ranged from two, at *1aac3b*, to 17, at *1tc1g*, with an average value of 7.8 (±4.6 SD).

**Figure 2:**
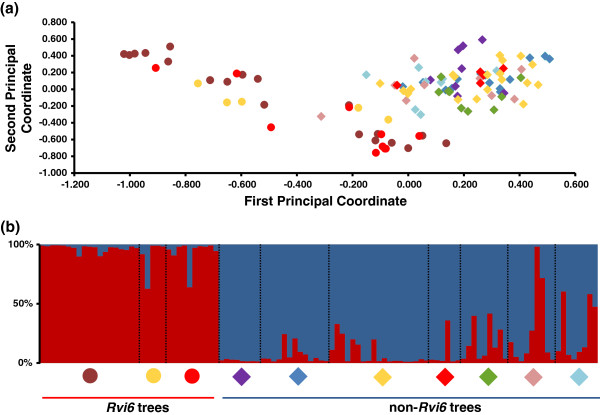
**The principal coordinates and STRUCTURE assignment analyses for the 106 *V. inaequalis *haplotypes. (a)** The first and second axes of the PCoA represent 27.9% and 18.7% of the total inertia. The strains collected from non-*Rvi6* hosts are represented by a diamond: *M. sieversii* (blue), *M. sylvestris* (purple), *M. coronaria* (green), *M. x purpurea* (light blue), *M. baccata* (yellow), *M. ioensis* (red) and *M. x zumi* (pink). The strains collected from *Rvi6* hosts are represented by a circle: *M. x floribunda* (brown), *M. baccata* (yellow) and *M. ioensis* (red). **(b)** Haplotypes are represented by a bar partitioned into *K = 2* segments that represent the haplotype’s estimated membership fractions calculated by STRUCTURE for each of the two clusters.

PCoA analysis separated *V. inaequalis* strains into three distinct groups with the first and second axes representing 27.9% and 18.7% of total inertia, respectively. The first axis obviously separated strains into two main groups: group 1 containing strains expressing a low score (< −0.4), and group 2 (> −0.4). The second principal coordinate also divided group 2 into two subgroups (“subgroup 2a” for negative values and “subgroup 2b” for positive values). Because the major resistance gene *Rvi6*, introgressed from the clone *Malus x floribunda* 821, is known to exert a high selective pressure on *V. inaequalis* populations [18], we evaluated the presence of this gene in all hosts sampled in this study (Additional file 1: Table S1). We showed that all accessions sampled on non-*Rvi6* hosts, *M. sieversii*, *M. sylvestris*, *M. baccata, M. coronaria, M. x zumi* (except for one strain) and *M. x purpurea*, were grouped into the single and close subgroup 2b (diamonds; Figure 2a). All strains sampled on the *Rvi6* trees, *M. x floribunda*, *M. ioensis* and *M. baccata*, are grouped into two different clusters, group 1 and subgroup 2a (circles; Figure 2a).

Traces of host specialisation within the *V. inaequalis* population were also assessed with the assignment method implemented in STRUCTURE. The clustering algorithm supported two clusters. Admixture coefficients for the best partitioning (K = 2) of all haplotypes were reported (Figure 2b). Strains assigned to one cluster (red; Figure 2b) were sampled on hosts carrying the *Rvi6* resistance gene, except for one strain isolated on *M. x zumi* (MAL0964), which would very likely be a first-generation migrant (Additional file 1: Table S3). Moreover, STRUCTURE detected 15 individuals with a Q admixture proportion to the first cluster of between 0.2 and 0.8 (14% of the strains), suggesting a substantial level of gene flow between the two clusters.

Additionally, AMOVA reveals that the two groups were significantly different (Φ_PT_ = 0.137; p = 0.01; Φ_PT_ is an analogue of F_ST_ that represents genetic diversity within and among populations [30]). Taken together, the analyses revealed two distinct subpopulations with some putative hybrids, which is indicative of gene flow between the two subpopulations.

For subsequent analyses, strains that were collected from hosts carrying the *Rvi6* gene were labelled “vir*Rvi6*”, whereas strains collected from non-*Rvi6* hosts were labelled “avr*Rvi6”* (Additional file 1: Table S3).

### No selection signature was detected within each differentiated subpopulation

To check whether the loci are neutral or targeted by natural selection, the distribution of F_ST_ against the probability to be under selective pressure was simulated with BayeScan software [32]. Considering the two previously detected subpopulations, the posterior probability revealed no marker plotted outside the 95% confidence interval constructed (data not shown). No SSR and AFLP loci were subject to selection, but instead exhibited a moderate to high level of neutral differentiation (mean F_ST_ ± SE = 0.179 ± 0.057). All markers were then considered as neutral and were used for all subsequent analyses.

#### No evidence of additional genetic structure related to the host

**Figure 3:**
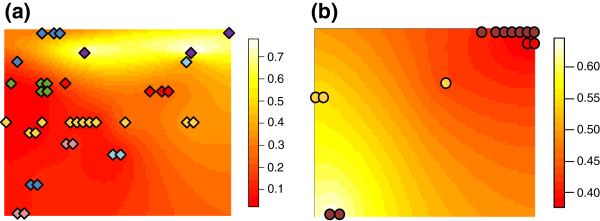
**The spatial interpolation map showing differences in the admixture population derived from the TESS assignment to two different clusters.** Given the two assumed clusters, the results for the avr*Rvi6* dataset **(a)** and the vir*Rvi6* dataset **(b)** are only shown for the first cluster assignment. The strains collected from non-*Rvi6* hosts are represented by a diamond: *M. sieversii* (blue), *M. sylvestris* (purple), *M. coronaria* (green), *M. x purpurea* (light blue), *M. baccata* (yellow), *M. ioensis* (red) and *M. x zumi* (pink). The strains collected from *Rvi6* hosts are represented by a circle: *M. x floribunda* (brown), *M. baccata* (yellow) and *M. ioensis* (red).

Considering these two subpopulations, molecular diversity indices revealed a higher diversity in the avr*Rvi6* than in the vir*Rvi6* subpopulation (Additional file 1: Table S4). Each dataset was then reanalysed under a clustering programme (STRUCTURE) to assess host-specificity within each subpopulation. Concerning the avr*Rvi6* subpopulation, application of Evanno’s method indicated two clusters. Individual assignments to the two clusters indicated that this genetic structure was not correlated to the host species (Additional file 2: Figure S1a). Subsequently, the avr*Rvi6* subpopulation was analysed by another clustering method (TESS), taking the spatial distribution of samples within the orchard into account. Simulations with Kmax = 2 for the avr*Rvi6* subpopulation indicated a spatial structure (Figure 3a). The posterior probability of an individual belonging to cluster 1 gradually increased with distance (Figure 3a). Sampling of strains on *M. baccata* trees along a line transect (the yellow diamonds in ranks 10 and 11; Figure 1) allowed us to validate the observed pattern of spatial structure (Figure 3a). Strain membership to cluster 1 gradually decreased from the left side of the rank to the right one along this transect, thus strengthening the idea that the main structure was spatial.

Concerning the vir*Rvi6* subpopulation, applications of a clustering method (STRUCTURE) and Evanno’s procedure revealed three clusters with a clear-cut Δk peak observed for K = 3. However, the three clusters in the vir*Rvi6* subpopulation were not correlated to the host species (Additional file 2: Figure S1b). For example, strains sampled on *M. x floribunda* were mainly assigned to each of the three clusters. Furthermore, the posterior probability of vir*Rvi6* samples belonging to cluster 1 inferred by TESS gradually decreased with distance. Genetic variation of strains sampled on *Rvi6* trees along a line transect also reflected a gradual decrease of membership to cluster 1 with distance (Figure 3b). The assignment of individuals to a higher value of K clusters (TESS assignment to three or four different clusters; Additional file 3: Figure S2) did not reveal any additional substructure linked to a particular host species.

Uses of Mantel tests using the Meirmans procedure [43] reinforced the interpretation that population genetic structure within each subpopulation is spatial rather than hierarchical. Indeed, Mantel statistics (*r*) are high and significant when spatial autocorrelation is explicitly tested, i.e., when testing the association between a matrix of genetic distance and a matrix of geographic distance (avr*Rvi6*: r = 0.14, p < 0.01; vir*Rvi6*: r = 0.15, p < 0.001; first three rows in Additional file 1: Table S5). On the contrary, testing the association between the matrix of genetic distances and a model of the matrix of cluster membership with the matrix of geographical distance as a covariate leads to lower *r* values and non-significant tests (avr*Rvi6*: r = 0.058, p = 0.051; vir*Rvi6*: r = 0.032, p = 0.342; last row, in Additional file 1: Table S5). This considerably strengthens our finding that the genetic structure within each subpopulation was mainly spatial.

### Detection of an IBD pattern within each subpopulation

**Figure 4:**
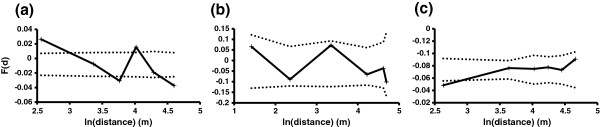
**Correlograms of mean kinship coefficients F(d) among pairs of isolates as a function of natural logarithm distance.** The analyses were performed for the avr*Rvi6* dataset **(a)**, vir*Rvi6* dataset **(b)**, and for the mean kinship in pairwise comparisons between avr*Rvi6* vs. vir*Rvi6* strains **(c)**. For each correlogram, mean kinship coefficients F(d) were estimated for each of the six different distance classes.

The presence of an IBD pattern was then tested within each subpopulation using two methods based on autocorrelograms [47] and variograms of gene diversity [46]. Correlograms based on kinship analyses showed a significant IBD pattern across the avr*Rvi6* subpopulation $\left(\overset{⌢}{b}=-0.0265\pm 0.0053\;r^{2}=0.0222,\;p=0\right)$ (Figure 4a). A non-significant IBD pattern (p = 0.085) was detected among the vir*Rvi6* strains $\left(\overset{⌢}{b}=-0.0307\pm 0.0080,\;r^{2}=0.0157\right)$ (Figure 4b) and between avr*Rvi6* vs. vir*Rvi6* individual pair comparisons (p = 0.99; Figure 4c) Moreover, we observed a global increase of gene diversity with distance (Additional file 4: Figures S3a and b). For the two subpopulations, gene diversity significantly increased between the first and second distance classes. The low gene diversity observed within each first class was never observed after random permutations of the dataset, which indicated a strong and fine-scale IBD pattern. These signals were lost after 20 metres in both cases (Additional file 4: Figures S3a and b), while no IBD pattern was detected between avr*Rvi6* vs. vir*Rvi6* individual pair comparisons (Additional file 4: Figure S3c).

## Discussion

Balance between selection and migration is crucial for the maintenance of genetic structure over time. On the one hand, Giraud *et al.*[7,8] reported that in some cases, host plants induce disruptive selective pressures on pathogen populations that are so high that gene flow is impeded, even if resident and new adapted populations are in sympatry. On the other hand, gene flow is particularly efficient in pathogens because of large population sizes and high fecundity, which is expected to facilitate population homogenisation. In this article, we studied the counteracting effects of the two forces by analysing the genetic structure of apple scab populations in a single orchard composed of different *Malus* species. We showed that the presence of the *Rvi6* resistance gene in *Malus* accessions divides the *V. inaequalis* population into two subpopulations. Within each subpopulation, an IBD pattern was detected at the orchard scale.

### Adaptations to resistance genes

Since wild apple species carry many scab resistance genes [15,50], we assumed that several host selective pressures exerted on *V. inaequalis* populations would be revealed in this study, as already demonstrated with the *Rvi6* gene [18]. The STRUCTURE clustering method revealed only two subpopulations: one subpopulation sampled from trees carrying the *Rvi6* gene and a second one infecting non-*Rvi6* trees, the distinction between these two clusters was also visible in the results of the PCoA. This population split was previously described in commercial orchards between infected *M. x domestica* cultivars carrying or not carrying the *Rvi6* gene [18-20]. Surprisingly, this study revealed that the sole presence of the *Rvi6* gene in the different wild *Malus* sp. induced an identical split in *V. inaequalis* populations, despite the very likely presence of other resistance genes in the diverse genetic background of the sampled accessions.

Evidence of admixture between the two subpopulations suggested that strains from the vir*Rvi6* subpopulation were able to infect non-*Rvi6* cultivars. However, admixture between the two subpopulations remained quite low, suggesting the existence of pre-zygotic and/or post-zygotic barriers to gene flow. Two different hypotheses can therefore be proposed: (i) the virulence cost (i.e., fitness cost on susceptible cultivars associated with a mutation to virulence) was strong enough to generate a strong pre-zygotic genetic barrier sufficient to isolate the vir*Rvi6* subpopulation from avr*Rvi6* by itself [7,18]; and (ii) avr*Rvi6* and vir*Rvi6* subpopulations were much more divergent than previously suspected and accumulated genetic barriers to gene flow other than the sole mutation at the avirulent locus. Divergence time estimation of these two subpopulations, associated with an investigation of divergent loci that impede free gene flow, is currently underway in the laboratory to test this last hypothesis.

No significant population genetic structure was detected within each subpopulation. Although different clusters were detected by STRUCTURE within each subpopulation, we did not find evidence of correlations between inferred clusters and host species. Given that *Rvi6* is the main resistance gene used in apple breeding programmes [51,52] and that no resistance gene other than *Rvi6* has been cloned in apple to date, genotyping other *Malus* resistance genes was not possible. On the one hand, we cannot exclude the existence of other structuring resistance factors shared by accessions belonging to different species. On the other hand, TESS assignments and map interpolations did reveal that the clusters detected by STRUCTURE in each subpopulation were more likely due to geographic discontinuities in the sampling scheme along the isolation gradient by distance than to other structuring factors related to host resistance genes. Furthermore, applications of partial Mantel tests using the Meirmans procedure [43] within each subpopulation reinforced our finding that structure was mainly spatial. Given the IBD, the sampling artefact is probably more consistent with detected clusters than other structuring factors.

### IBD and dispersal abilities

The SGS analyses performed within each avr*Rvi6* and vir*Rvi6* subpopulation highlighted a decrease of mean kinship between strains when the distance increased. First, if host adaptations other than *Rvi6* existed in this orchard, restricted gene flow between structured populations would be detected. Conversely, IBD detection and the gradual assignment to a TESS cluster associated with distance suggested that strains easily shifted from one tree to another, regardless of the host species. Gene flow between individuals of each subpopulation was free, even if the neighbour host belonged to another species. Second, IBD detection across this orchard highlighted restricted *V. inaequalis* dispersal over space. Holb *et al.*[16] showed that the mean daily ascospore count in traps at 21 metres and 45 metres was approximately one-third and one-tenth of the sexual spores trapped at the source, respectively, highlighting that a substantial number of ascospores could travel at least 45 metres from the inoculum source. Lower dispersal abilities were previously reported by Kaplan [53] who observed that 99% of the spores are not able to spread more than 5 or 6 metres. Many factors can explain these dispersal distance differences, including orchard conditions and differences in wind direction and velocity (see [16]). In this study, we confirmed that dispersal of the greater part of *V. inaequalis* spores in orchards was intrinsically very restricted, which is in agreement with several other authors (e.g., [54-56]), who demonstrated that no scab lesion development was detected at 15 to 60 metres beyond an inoculum source. We do not dismiss the sporadic events of Long Distance Dispersal (LDD). However, based on a simulation study, Aylor [57] has shown that *V. inaequalis* ascospores cannot disperse in the air over more than 5 km, even under favourable weather conditions for the pathogen.

### Detection of signatures of natural selection

Comparing vir*Rvi6* and avr*Rvi6* subpopulations, none of the markers (AFLP, microsatellites) were detected as outliers using the Foll & Gaggiotti method [32]. Several hypotheses can support this lack of detection. First, if the number and the size of the genomic regions affected by selection were low, the sampling effort was probably insufficient to detect loci affected by divergent natural selection. Such a hypothesis is likely if recombination between the two subpopulations was efficient enough to reduce the size of the genomic region of high F_ST_, thus limiting high differentiation to the immediate genomic neighbourhood of the vir*Rvi6* locus. Second, our genome scan might not be powerful enough to detect loci under directional selection. Indeed, the genome scan was performed on a majority of AFLP markers (i.e., bi-allelic), by comparing only two subpopulations that, moreover, exhibited a significant population structure. A low number of alleles, a reduced number of populations and a strong population genetic structure are known to strongly reduce the power of outlier detection [32].

## Conclusions

In conclusion, we detected two genetic patterns of population structure in *V. inaequalis* in an orchard with numerous *Malus* species. The highly structuring effect of the presence or absence of a single resistance gene led to a split of the pathogen population into two subpopulations. Additionally, an IBD pattern was detected within each of these two subpopulations. Our work represents a rare example in pathogens where the dispersal evaluated by a fine scale IBD pattern appropriately fits with previously reported empirical field data [16,53]. Because many pathogen dispersal capabilities remain unclear, our validation of the IBD approach is particularly significant for epidemiologists and also has practical implications for plant breeders and landscape managers for the design of disease control strategies.

## Availability of supporting data

The dataset is available through the DRYAD data archive system (http://dx.doi.org/10.5061/dryad.970b3).

## Competing interests

The authors declare no competing interests.

## Authors’ contributions

The work presented here was carried out jointly with all of the authors. All of the authors defined the research theme. TL carried out the *V. inaequalis* laboratory experiments, analysed the data, interpreted the results and drafted the paper. FD performed the genotyping of *Malus* trees at the *Rvi6* locus. All of the authors participated in the critical revision of the manuscript and gave final approval of the article.

## Supplementary Material

Additional file 1: Table S1 The geographical and plant origins of the samples used in this study. The host species and accession number of the sampled tree, host localisation (rank, localisation inside the rank, X and Y coordinates) and alleles at the Rvi6 resistance locus were reported. The following information concerns the number of sampled strains (n). * An apple genotype was classified as carrying the Rvi6 locus (“1”) when all three alleles were detected (CH-Vf1: allele 159, AL07-SCAR: 480 bp fragment, Vfa2: 550 bp fragment), or when the Rvi6 allele sequence was known (Dunemann, pers. comm.), (“0”) if all three allele assays were negative, (“?”) if only one or two alleles were detected, or (“nd”) for missing data. **Table S2**: The genetic diversity for strains sampled on each tree species. n represents the number of strains collected from each host, Ka the number of haplotypes, and Hd the average gene diversity calculated according to Nei (1987) and estimated from clone-corrected datasets. **Table S3**: A list of strains grouped into each subpopulation. In both subpopulations, the origin of samples (tree species and genotype at the Rvi6 locus) is reported. For each strain, main membership to one of the two clusters inferred by STRUCTURE was also reported (1 for red and 2 for blue in Figure 2b). * An apple genotype was classified as carrying the Rvi6 locus (“yes”) when all three alleles were detected (CH-Vf1: allele 159, AL07-SCAR: 480 bp fragment, Vfa2: 550 bp fragment), or when the Rvi6 allele sequence was known (**), (“no”) if all three allele assays were negative, (“?”) if only one or two alleles were detected, or (“nd”) for missing data. **Table S4**: The genetic diversity for each subpopulation. n represents the number of strains in each subpopulation, Ka the number of unique haplotypes estimated for non-clone-corrected datasets, Hd the average gene diversity, and A the average number of alleles estimated from clone-corrected datasets. Hd was calculated according to Nei (1987). **Table S5**: Results of standard, stratified, and partial Mantel tests for avrRvi6 and virRvi6 subpopulations. Stars indicate the significance level: * p < 0.05, **p < 0.01, and ***p < 0.001. See Materials and Method section for explanation on Mantel tests and [43] for more details on the procedure. Click here for file

Additional file 2: Figure S1 STRUCTURE individual assignments of K = 2 or K = 3 clusters inferred for avr*Rvi6* (a) and vir*Rvi6* (b) subpopulations. Each haplotype is represented by a bar partitioned into *K = 2* or K = 3 segments that represent the haplotype’s estimated membership fractions in each of the two or three clusters. For each fungal haplotype, the *Malus* species where strains were sampled is indicated below each chart. Click here for file

Additional file 3: Figure S2 The spatial interpolation map showing differences in the admixture population derived from the TESS assignment to three or four different clusters within each subpopulation. The interpolations for the avr*Rvi6* dataset, assuming Kmax = 3 (a) and Kmax =4, and for vir*Rvi6* dataset with Kmax = 3 (c) and Kmax = 4 (d), are shown. The strains collected from non-*Rvi6* hosts are represented by a diamond: *M. sieversii* (blue), *M. sylvestris* (purple), *M. coronaria* (green), *M. x purpurea* (light blue), *M. baccata* (yellow), *M. ioensis* (red) and *M. x zumi* (pink). The strains collected from *Rvi6* hosts are represented by a circle: *M. x floribunda* (brown), *M. baccata* (yellow) and *M. ioensis* (red). Click here for file

Additional file 4: Figure S3 The variograms of gene diversity computed for the avr*Rvi6* subpopulation (a), the vir*Rvi6* subpopulation (b) and avr*Rvi6* vs. vir*Rvi6* pairwise comparisons (c). Ninety-five percent confidence interval limits were plotted as dotted lines. Gene diversity was estimated for different distance classes for each variogram: 15 for the avr*Rvi6* subpopulation, five for the vir*Rvi6* subpopulation, and 15 for avr*Rvi6* vs. vir*Rvi6* subpopulation pairwise comparisons. Click here for file
