# Supplementary material for: The genetic structure of a Venturia inaequalis population in a heterogeneous host population composed of different Malus species
Source: BMC Evol Biol. 2013 Mar 12;13:64. doi: 10.1186/1471-2148-13-64 (PMC3626921; doi:10.1186/1471-2148-13-64)
Supplement: Additional file 1: Table S1 — The geographical and plant origins of the samples used in this study. The host species and accession number of the sampled tree, host localisation (rank, localisation inside the rank, X and Y coordinates) and alleles at the Rvi6 resistance locus were reported. The following information concerns the number of sampled strains (n). * An apple genotype was classified as carrying the Rvi6 locus (“1”) when all three alleles were detected (CH-Vf1: allele 159, AL07-SCAR: 480 bp fragment, Vfa2: 550 bp fragment), or when the Rvi6 allele sequence was known (Dunemann, pers. comm.), (“0”) if all three allele assays were negative, (“?”) if only one or two alleles were detected, or (“nd”) for missing data. Table S2: The genetic diversity for strains sampled on each tree species. n represents the number of strains collected from each host, Ka the number of haplotypes, and Hd the average gene diversity calculated according to Nei (1987) and estimated from clone-corrected datasets. Table S3: A list of strains grouped into each subpopulation. In both subpopulations, the origin of samples (tree species and genotype at the Rvi6 locus) is reported. For each strain, main membership to one of the two clusters inferred by STRUCTURE was also reported (1 for red and 2 for blue in Figure 2b). * An apple genotype was classified as carrying the Rvi6 locus (“yes”) when all three alleles were detected (CH-Vf1: allele 159, AL07-SCAR: 480 bp fragment, Vfa2: 550 bp fragment), or when the Rvi6 allele sequence was known (**), (“no”) if all three allele assays were negative, (“?”) if only one or two alleles were detected, or (“nd”) for missing data. Table S4: The genetic diversity for each subpopulation. n represents the number of strains in each subpopulation, Ka the number of unique haplotypes estimated for non-clone-corrected datasets, Hd the average gene diversity, and A the average number of alleles estimated from clone-corrected datasets. Hd was calculated according to Nei (1987). Table S5: Result [file 1471-2148-13-64-S1.doc]

Supplementary data, Table 1: The geographical and plant origins of the samples used in this study. The host species and accession number of the sampled tree, host localisation (rank, localisation inside the rank, X and Y coordinates) and alleles at the Rvi6 resistance locus were reported. The following information concerns the number of sampled strains (n). * An apple genotype was classified as carrying the Rvi6 locus (“1”) when all three alleles were detected (CH-Vf1: allele 159, AL07-SCAR: 480 bp fragment, Vfa2: 550 bp fragment), or when the Rvi6 allele sequence was known (Dunemann, pers. comm.), (“0”) if all three allele assays were negative, (“?”) if only one or two alleles were detected, or (“nd”) for missing data.

| Host | Acc. Numb. | X coordinate | Y coordinate | *Rvi6* tree | n |
| --- | --- | --- | --- | --- | --- |
| *M. sieversii* | MAL0946 | 21 | 85.5 | 0 | 1 |
|  | MAL0948 | 28 | 85.5 | 0 | 3 |
|  | MAL0949 | 31.5 | 85.5 | 0 | 3 |
|  | MAL0729 | 7 | 72 | 0 | 1 |
|  | MAL0789 | 14 | 18 | 0 | 2 |
|  | MAL0789 | 17.5 | 18 | 0 | 4 |
| All *M. sieversii* | | | |  | 14 |
|  |  |  |  |  |  |
| *M. sylvestris* | MAL0907 | 129.5 | 85.5 | 0 | 1 |
|  | MAL0925 | 45.5 | 76.5 | 0 | 3 |
|  | MAL0927 | 108.5 | 76.5 | 0 | 5 |
| All *M. sylvestris* | | | |  | 9 |
|  |  |  |  |  |  |
| *M. baccata* | MAL0043 | 0 | 45 | 0 | 2 |
|  | MAL0373 | 21 | 45 | 0 | 1 |
|  | MAL0328 | 38.5 | 45 | 0 | 1 |
|  | MAL0377 | 42 | 45 | 0 | 2 |
|  | MAL0377 | 45.5 | 45 | 0 | 2 |
|  | MAL0396 | 49 | 45 | 0 | 2 |
|  | MAL0396 | 52.5 | 45 | 0 | 2 |
|  | MAL0324 | 66.5 | 45 | 1 | 2 |
|  | MAL0458 | 70 | 45 | 0 | 1 |
|  | MAL0780 | 105 | 45 | 0 | 2 |
|  | MAL0780 | 108.5 | 45 | 0 | 1 |
|  | MAL0421 | 17.5 | 40.5 | 0 | 1 |
|  | MAL0421 | 14 | 40.5 | 0 | 2 |
|  | MAL0156 | 10.5 | 40.5 | 1 | 1 |
|  | MAL0156 | 7 | 40.5 | 1 | 2 |
| All *M. baccata* | | | |  | 24 |

Supplementary data, Table 1: The geographical and plant origins of the samples used in this study (continued).

| Host | Acc. Numb. | X coordinate | Y coordinate | *Rvi6* tree | n |
| --- | --- | --- | --- | --- | --- |
| *M. ioensis* | MAL0298 | 35 | 63 | 0 | 2 |
|  | MAL0134 | 84 | 58.5 | 0 | 1 |
|  | MAL0343 | 91 | 58.5 | 0 | 1 |
|  | MAL0343 | 94.5 | 58.5 | 0 | 1 |
|  | MAL0330 | 105 | 58.5 | 1 | 5 |
|  | MAL0330 | 108.5 | 58.5 | 1 | 5 |
| All *M. ioensis* | | | |  | 15 |
|  |  |  |  |  |  |
| *M. coronaria* | MAL0350 | 3.5 | 63 | 0 | 2 |
|  | MAL0734 | 21 | 63 | nd | 1 |
|  | MAL0734 | 24.5 | 63 | nd | 1 |
|  | MAL0340 | 21 | 58.5 | 0 | 3 |
|  | MAL0340 | 24.5 | 58.5 | 0 | 3 |
| All *M. coronaria* | | | |  | 10 |
|  |  |  |  |  |  |
| *M. x zumi* | MAL0364 | 35 | 36 | 0 | 1 |
|  | MAL0364 | 38.5 | 36 | 0 | 3 |
|  | MAL0964 | 7 | 4.5 | ? | 2 |
|  | MAL0964 | 10.5 | 4.5 | ? | 3 |
| All *M. x zumi* | | | |  | 9 |
|  |  |  |  |  |  |
| *M. x purpurea* | MAL0268 | 105 | 72 | 0 | 4 |
|  | MAL0796 | 63 | 31.5 | ? | 5 |
|  | MAL0796 | 66.5 | 31.5 | ? | 2 |
|  | MAL0728 | 49 | 31.5 | 0 | 1 |
| All *M. x purpurea* | | | |  | 12 |
|  |  |  |  |  |  |
| *M. x floribunda 821* | MAL0989 | 14 | 0 | 1 | 4 |
|  | MAL0989 | 17.5 | 0 | 1 | 5 |
| *Other M. x floribunda* | MAL0054 | 84 | 63 | 1 | 1 |
|  | MAL0401 | 91 | 63 | 1 | 3 |
|  | MAL0401 | 94.5 | 63 | 1 | 2 |
|  | MAL0359 | 98 | 63 | 1 | 1 |
|  | MAL0359 | 101.5 | 63 | 1 | 3 |
|  | MAL0012 | 105 | 63 | 1 | 1 |
|  | MAL0012 | 108.5 | 63 | 1 | 1 |
| All *M. x floribunda* | | | |  | 21 |
|  |  |  |  |  |  |
| **All sampled hosts** | | | |  | **114** |

# Supplementary data, Table 2: The genetic diversity for strains sampled on each tree species. n represents the number of strains collected from each host, K_a_ the number of haplotypes, and H_d_ the average gene diversity calculated according to Nei (1987) and estimated from clone-corrected datasets.

| Strains collected on | *n* | *K_a_* | H_d_ |
| --- | --- | --- | --- |
| *M. x floribunda 821* | 9 | 7 | 0.17 |
| *Other M. x floribunda* | 12 | 12 | 0.26 |
| *M. sylvestris* | 9 | 8 | 0.22 |
| *M. sieversii* | 14 | 14 | 0.33 |
| *M. baccata* | 24 | 24 | 0.33 |
| *M. coronaria* | 10 | 9 | 0.28 |
| *M. x purpurea* | 12 | 8 | 0.28 |
| *M. x zumi* | 9 | 9 | 0.32 |
| *M. ioensis* | 15 | 15 | 0.23 |
| All | 114 | 106 | 0.27 |

Supplementary data, Table 3: A list of strains grouped into each subpopulation. In both subpopulations, the origin of samples (tree species and genotype at the *Rvi6* locus) is reported. For each strain, main membership to one of the two clusters inferred by STRUCTURE was also reported (1 for red and 2 for blue in Fig. 2b). * An apple genotype was classified as carrying the *Rvi6* locus (“yes”) when all three alleles were detected (CH-Vf1: allele 159, AL07-SCAR: 480 bp fragment, Vfa2: 550 bp fragment), or when the *Rvi6* allele sequence was known (**), (“no”) if all three allele assays were negative, (“?”) if only one or two alleles were detected, or (“nd”) for missing data.

| Subpop. | Strain | Host species | *Rvi6* tree* | Structure cluster |
| --- | --- | --- | --- | --- |
| vir*Rvi6* | 08M209 | *M. x floribunda 821* | **yes** | 1 |
|  | 08M395a | *M. x floribunda 821* | **yes** | 1 |
|  | 08M395b | *M. x floribunda 821* | **yes** | 1 |
|  | 08M396 | *M. x floribunda 821* | **yes** | 1 |
|  | 08M397 | *M. x floribunda 821* | **yes** | 1 |
|  | 08M211 | *M. x floribunda 821* | **yes** | 1 |
|  | 08M237 | *M. x floribunda 821* | **yes** | 1 |
|  | 08M239 | *Other M. x floribunda* | **yes** | 1 |
|  | 08M243 | *Other M. x floribunda* | **yes** | 1 |
|  | 08M401b | *Other M. x floribunda* | **yes** | 1 |
|  | 08M406 | *Other M. x floribunda* | **yes** | 1 |
|  | 08M407 | *Other M. x floribunda* | **yes** | 1 |
|  | 08M408 | *Other M. x floribunda* | **yes** | 1 |
|  | 08M409 | *Other M. x floribunda* | **yes** | 1 |
|  | 08M410 | *Other M. x floribunda* | **yes** | 1 |
|  | 08M411 | *Other M. x floribunda* | **yes** | 1 |
|  | 08M412 | *Other M. x floribunda* | **yes** | 1 |
|  | 08M214 | *Other M. x floribunda* | **yes** | 1 |
|  | 08M233 | *Other M. x floribunda* | **yes** | 1 |
|  | 08M162a | *M. baccata* | **yes** | 1 |
|  | 08M162b | *M. baccata* | **yes** | 1 |
|  | 08M175 | *M. baccata* | **yes** | 1 |
|  | 08M177 | *M. baccata* | **yes** | 1 |
|  | 08M178 | *M. baccata* | **yes** | 1 |
|  | 08M199a | *M. ioensis* | **yes**** | 1 |
|  | 08M199b | *M. ioensis* | **yes**** | 1 |
|  | 08M200a | *M. ioensis* | **yes**** | 1 |
|  | 08M200b | *M. ioensis* | **yes**** | 1 |
|  | 08M2010 | *M. ioensis* | **yes**** | 1 |
|  | 08M202b | *M. ioensis* | **yes**** | 1 |
|  | 08M203 | *M. ioensis* | **yes**** | 1 |
|  | 08M204 | *M. ioensis* | **yes**** | 1 |
|  | 08M205 | *M. ioensis* | **yes**** | 1 |
|  | 08M206 | *M. ioensis* | **yes**** | 1 |

Supplementary data, Table 3: A list of strains grouped into each subpopulation (continued).

| Subpop. | Strain | Host species | *Rvi6* tree* | Structure cluster |
| --- | --- | --- | --- | --- |
| avr*Rvi6* | 08M116 | *M. sylvestris* | no | 2 |
|  | 08M128 | *M. sylvestris* | no | 2 |
|  | 08M129 | *M. sylvestris* | no | 2 |
|  | 08M130b | *M. sylvestris* | no | 2 |
|  | 08M133a | *M. sylvestris* | no | 2 |
|  | 08M133c | *M. sylvestris* | no | 2 |
|  | 08M134a | *M. sylvestris* | no | 2 |
|  | 08M135 | *M. sylvestris* | no | 2 |
|  | 08M91 | *M. sieversii* | no | 2 |
|  | 08M92 | *M. sieversii* | no | 2 |
|  | 08M94a | *M. sieversii* | no | 2 |
|  | 08M94b | *M. sieversii* | no | 2 |
|  | 08M95 | *M. sieversii* | no | 2 |
|  | 08M96 | *M. sieversii* | no | 2 |
|  | 08M97 | *M. sieversii* | no | 2 |
|  | 08M98a | *M. sieversii* | no | 2 |
|  | 08M108 | *M. sieversii* | no | 2 |
|  | 08M110 | *M. sieversii* | no | 2 |
|  | 08M111 | *M. sieversii* | no | 2 |
|  | 08M112 | *M. sieversii* | no | 2 |
|  | 08M113 | *M. sieversii* | no | 2 |
|  | 08M114 | *M. sieversii* | no | 2 |
|  | 08M136 | *M. baccata* | no | 2 |
|  | 08M137 | *M. baccata* | no | 2 |
|  | 08M146 | *M. baccata* | no | 2 |
|  | 08M153 | *M. baccata* | no | 2 |
|  | 08M154 | *M. baccata* | no | 2 |
|  | 08M155 | *M. baccata* | no | 2 |
|  | 08M156 | *M. baccata* | no | 2 |
|  | 08M157 | *M. baccata* | no | 2 |
|  | 08M158 | *M. baccata* | no | 2 |
|  | 08M159 | *M. baccata* | no | 2 |
|  | 08M160 | *M. baccata* | no | 2 |
|  | 08M161 | *M. baccata* | no | 2 |
|  | 08M163 | *M. baccata* | no | 2 |
|  | 08M167 | *M. baccata* | no | 2 |
|  | 08M168 | *M. baccata* | no | 2 |
|  | 08M170 | *M. baccata* | no | 2 |
|  | 08M172 | *M. baccata* | no | 2 |
|  | 08M173 | *M. baccata* | no | 2 |
|  | 08M174 | *M. baccata* | no | 2 |
|  | 08M180b | *M. ioensis* | no | 2 |
|  | 08M181a | *M. ioensis* | no | 2 |
|  | 08M185 | *M. ioensis* | no | 2 |
|  | 08M192 | *M. ioensis* | no | 2 |
|  | 08M195 | *M. ioensis* | no | 2 |
|  | 08M248b | *M. coronaria* | no | 2 |
|  | 08M249b | *M. coronaria* | no | 2 |
|  | 08M294a | *M. coronaria* | nd | 2 |
|  | 08M297b | *M. coronaria* | nd | 2 |

Supplementary data, Table 3: A list of strains grouped into each subpopulation (continued).

| Subpop. | Strain | Host species | *Rvi6* tree* | Structure cluster |
| --- | --- | --- | --- | --- |
|  | 08M251b | *M. coronaria* | no | 2 |
|  | 08M251c | *M. coronaria* | no | 2 |
|  | 08M256a | *M. coronaria* | no | 2 |
|  | 08M257b | *M. coronaria* | no | 2 |
|  | 08M258 | *M. coronaria* | no | 2 |
|  | 08M261 | *M. x zumi* | no | 2 |
|  | 08M267 | *M. x zumi* | no | 2 |
|  | 08M268 | *M. x zumi* | no | 2 |
|  | 08M269 | *M. x zumi* | no | 2 |
|  | 08M271 | *M. x zumi* | no | 2 |
|  | 08M273 | *M. x zumi* | ? | 1 |
|  | 08M275 | *M. x zumi* | ? | 2 |
|  | 08M278 | *M. x zumi* | ? | 2 |
|  | 08M279 | *M. x zumi* | ? | 2 |
|  | 08M306 | *M. x purpurea* | no | 2 |
|  | 08M307 | *M. x purpurea* | no | 2 |
|  | 08M309a | *M. x purpurea* | no | 2 |
|  | 08M309b | *M. x purpurea* | no | 2 |
|  | 08M315 | *M. x purpurea* | ? | 2 |
|  | 08M321 | *M. x purpurea* | ? | 2 |
|  | 08M327 | *M. x purpurea* | ? | 2 |
|  | 08M329 | *M. x purpurea* | ? | 2 |

# Supplementary data, Table 4: The genetic diversity for each subpopulation. n represents the number of strains in each subpopulation, K_a_ the number of unique haplotypes estimated for non-clone-corrected datasets, H_d_ the average gene diversity, and A the average number of alleles estimated from clone-corrected datasets. H_d_ was calculated according to Nei (1987).

| Subpop. | *n* | *K_a_* | H_d_ | A |  |
| --- | --- | --- | --- | --- | --- |
| avr*Rvi6* | 36 | 34 | 0.26 | 1.84 |  |
| vir*Rvi6* | 78 | 72 | 0.33 | 2.69 |  |
| All | 114 | 106 | 0.30 | 2.27 |  |
|  |  |  |  |  |  |

Supplementary data, Table 5: Results of standard, stratified, and partial Mantel tests for avr*Rvi6* and vir*Rvi6* subpopulations. Stars indicate the significance level: * *p<0.05, **p<* 0.01, and ***p<0.001. See Materials and Method section for explanation on Mantel tests and [43] for more details on the procedure.

| Matrix A | Matrix B | Adjustment | Mantel's *r* | |
| --- | --- | --- | --- | --- |
|  |  |  | avr*Rvi6* subpop. | vir*Rvi6* subpop. |
| Genetic | Geographic | None | 0.146*** | 0.150** |
| Genetic | Geographic | Stratified: permuted within clusters | 0.146*** | 0.150** |
| Genetic | Geographic | Partial: clusters as covariate | 0.145*** | 0.153** |
| Genetic | Clusters | Partial: geography as covariate | 0.058 | 0.032 |
