# Supplementary figures and images for: The genetic structure of a Venturia inaequalis population in a heterogeneous host population composed of different Malus species
Source: BMC Evol Biol. 2013 Mar 12;13:64. doi: 10.1186/1471-2148-13-64 (PMC3626921; doi:10.1186/1471-2148-13-64)

(a)

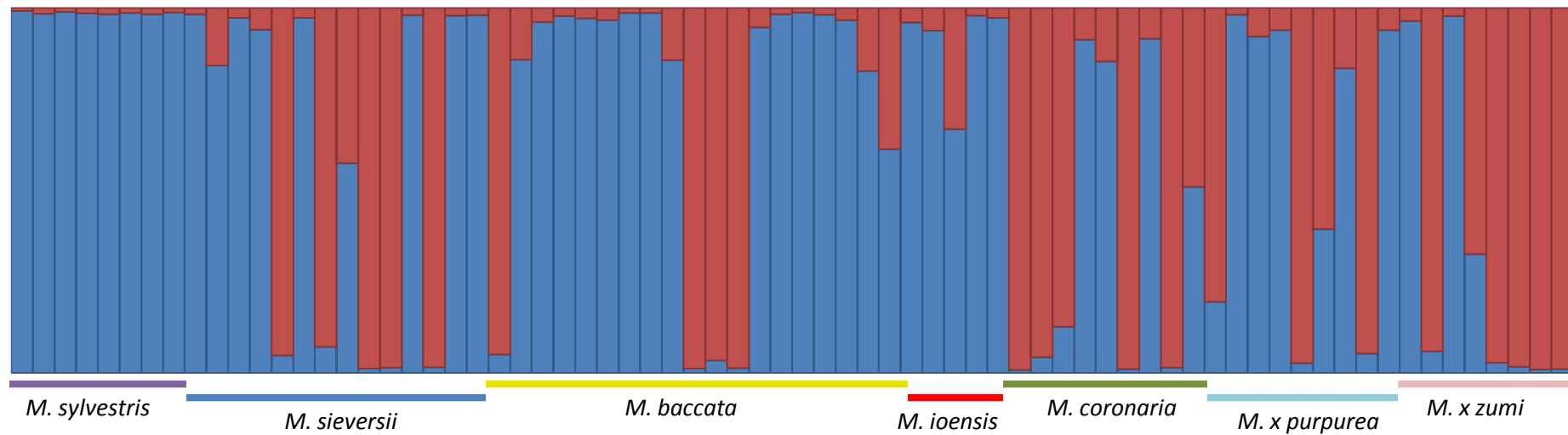

(b)

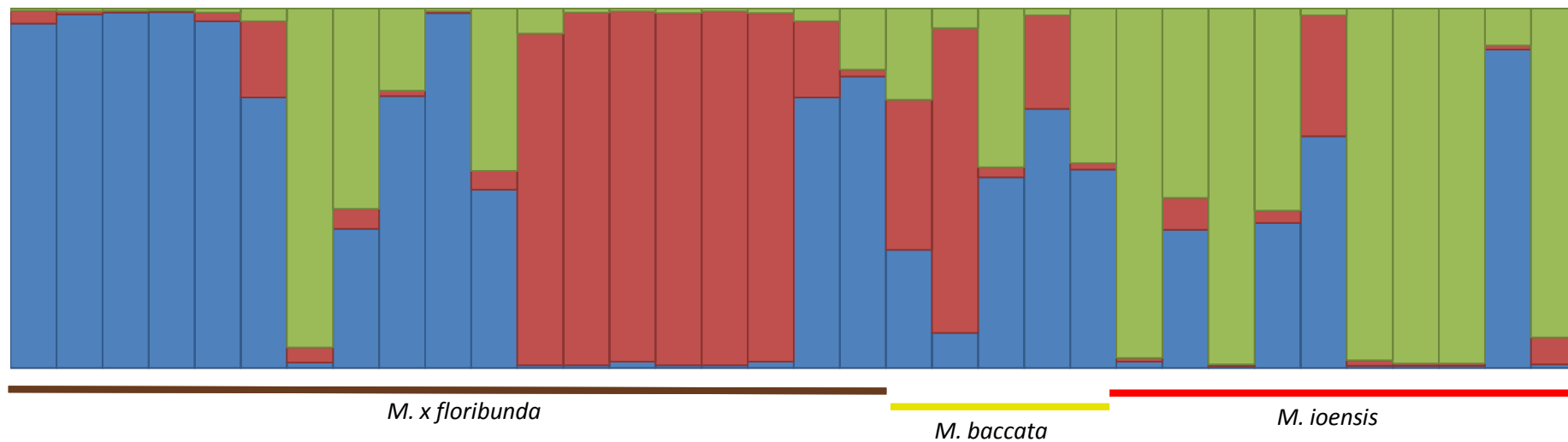

Supplement: Additional file 2: Figure S1 — STRUCTURE individual assignments of K = 2 or K = 3 clusters inferred for avrRvi6 (a) and virRvi6 (b) subpopulations. Each haplotype is represented by a bar partitioned into K = 2 or K = 3 segments that represent the haplotype’s estimated membership fractions in each of the two or three clusters. For each fungal haplotype, the Malus species where strains were sampled is indicated below each chart. [file 1471-2148-13-64-S2.pdf]

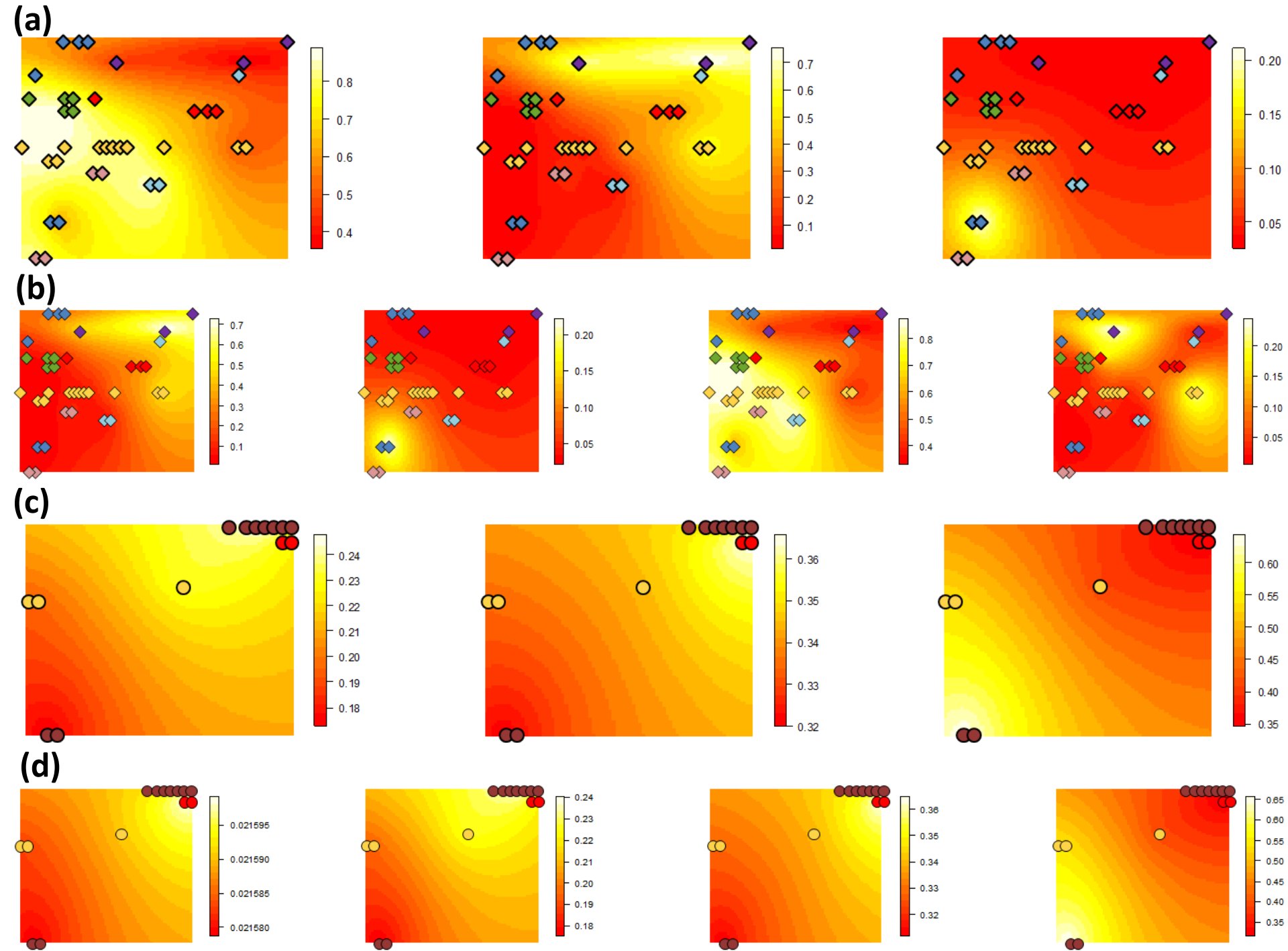

Supplement: Additional file 3: Figure S2 — The spatial interpolation map showing differences in the admixture population derived from the TESS assignment to three or four different clusters within each subpopulation. The interpolations for the avrRvi6 dataset, assuming Kmax = 3 (a) and Kmax =4, and for virRvi6 dataset with Kmax = 3 (c) and Kmax = 4 (d), are shown. The strains collected from non-Rvi6 hosts are represented by a diamond: M. sieversii (blue), M. sylvestris (purple), M. coronaria (green), M. x purpurea (light blue), M. baccata (yellow), M. ioensis (red) and M. x zumi (pink). The strains collected from Rvi6 hosts are represented by a circle: M. x floribunda (brown), M. baccata (yellow) and M. ioensis (red). [file 1471-2148-13-64-S3.pdf]

**(a)**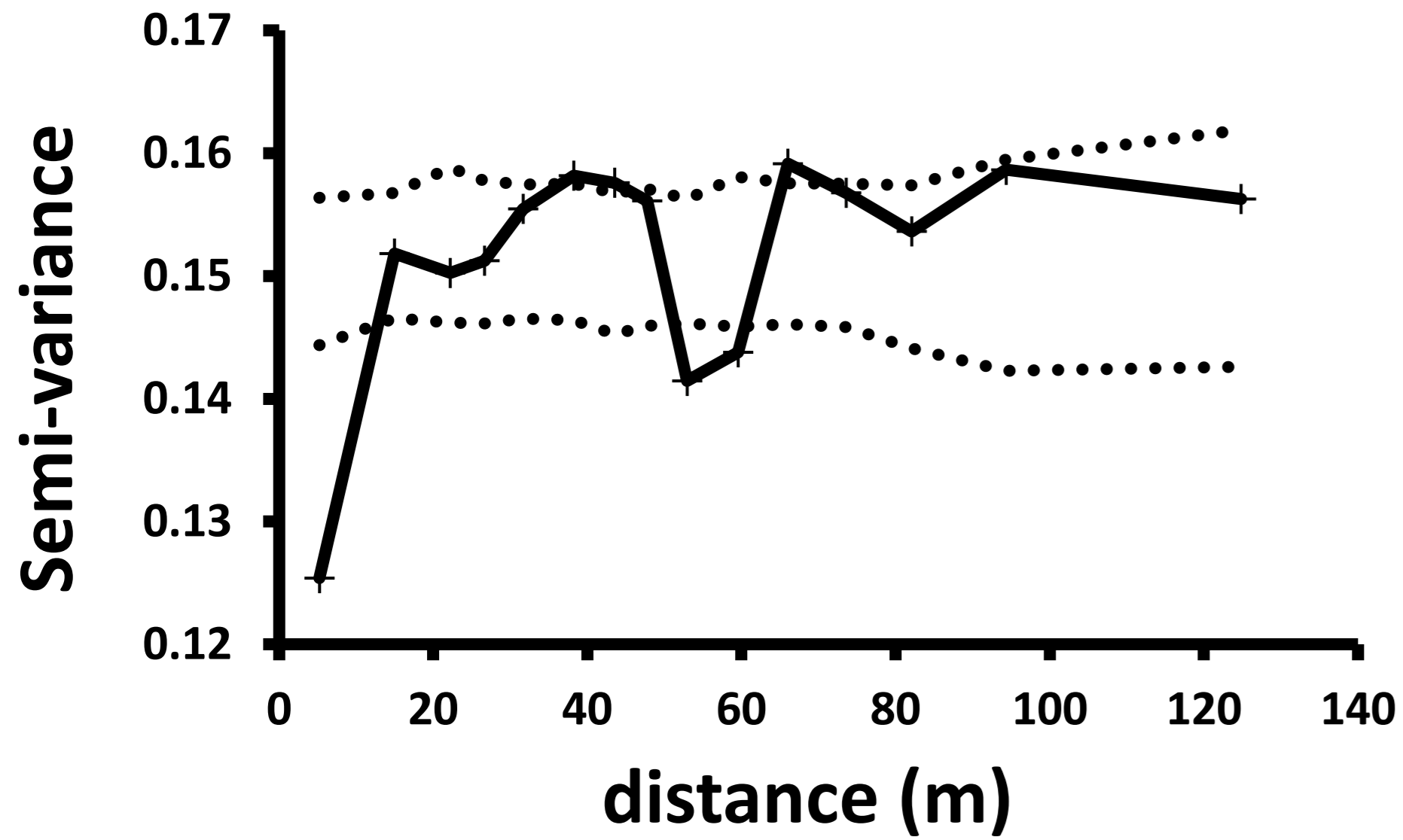**(b)**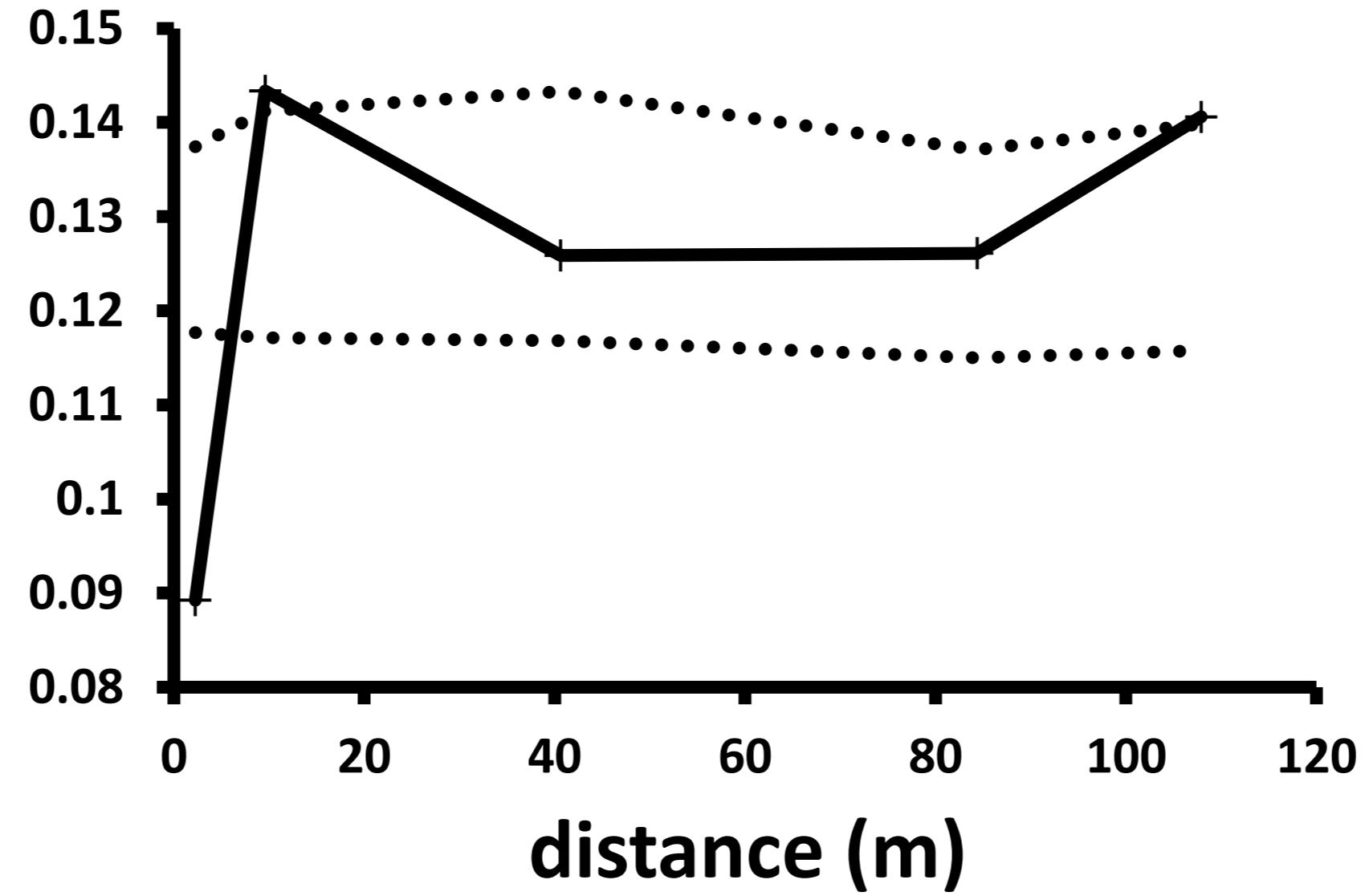**(c)**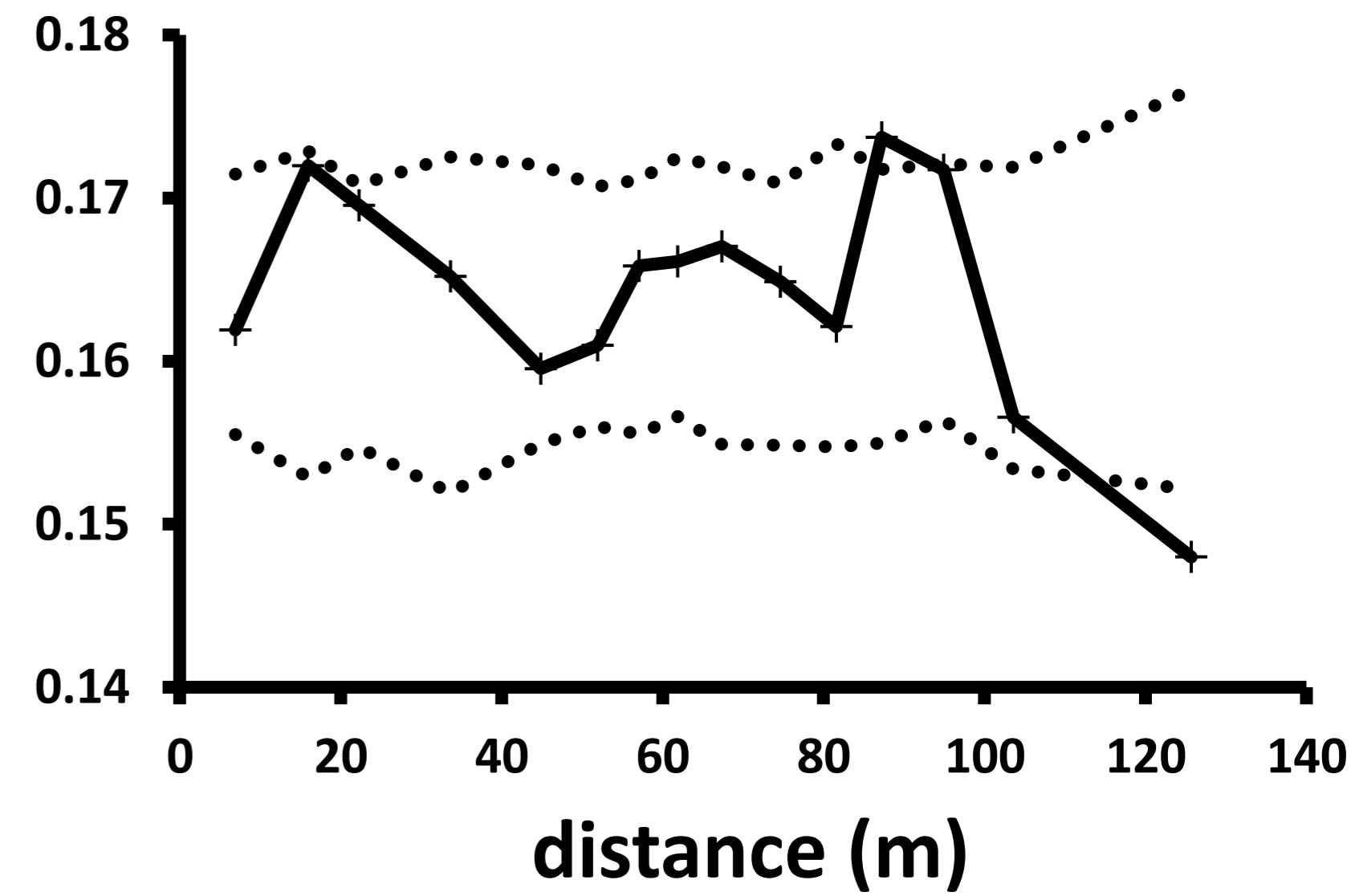

Supplement: Additional file 4: Figure S3 — The variograms of gene diversity computed for the avrRvi6 subpopulation (a), the virRvi6 subpopulation (b) and avrRvi6 vs. virRvi6 pairwise comparisons (c). Ninety-five percent confidence interval limits were plotted as dotted lines. Gene diversity was estimated for different distance classes for each variogram: 15 for the avrRvi6 subpopulation, five for the virRvi6 subpopulation, and 15 for avrRvi6 vs. virRvi6 subpopulation pairwise comparisons. [file 1471-2148-13-64-S4.pdf]
